# Drug/diet synergy for managing malignant astrocytoma in mice: 2-deoxy-D-glucose and the restricted ketogenic diet

**DOI:** 10.1186/1743-7075-5-33

**Published:** 2008-11-25

**Authors:** Jeremy Marsh, Purna Mukherjee, Thomas N Seyfried

**Affiliations:** 1 Biology Department, Boston College, Chestnut Hill, MA 02467, USA

## Abstract

**Background:**

Astrocytomas are largely dependent on glycolysis to satisfy their bioenergetic requirements for growth and survival. Therapies that target glycolysis can potentially manage astrocytoma growth and progression. Dietary restriction of the high fat/low carbohydrate ketogenic diet (KD-R) reduces glycolysis and is effective in managing experimental mouse and human astrocytomas. The non-metabolizable glucose analogue, 2-deoxy-D-glucose (2-DG), is a potent glycolytic inhibitor that can mimic effects of energy restriction both *in vitro *and *in vivo*, but can also produce adverse effects when administered at doses greater than 200 mg/kg. The goal here was to determine if low doses of 2-DG could act synergistically with the KD-R to better manage growth of the CT-2A malignant mouse astrocytoma.

**Methods:**

The therapeutic effect of a KD-R supplemented with a low dose of 2-DG (25 mg/kg) was examined in adult C57BL/6J mice bearing the syngeneic CT-2A malignant astrocytoma grown orthotopically. Mice were fed the standard unrestricted diet for the first 3 days after tumor implantation prior to their separation into one of four diet groups fed either a standard rodent diet in unrestricted amounts (SD-UR) or a KD-R with or without 2-DG for 10 days. The KD-R was restricted to reduce body weight by about 20%. 2-DG was initiated 6 days after tumor implantation and was continued for 7 days. Brain tumors were excised and weighed.

**Results:**

Energy intake, body weights, and CT-2A tumor weights were similar in the SD-UR and the SD-UR+2-2DG mouse groups over the dietary treatment period (days 3–13). Tumor weights were about 48% and 80% lower in the KD-R and in the KD-R+2-DG groups, respectively, than in the SD-UR group. Mouse health and vitality was better in the KD-R group than in the KD-R+2-DG group.

**Conclusion:**

Astrocytoma growth was reduced more in the KD-R mouse group supplemented with 2-DG than in the mouse groups receiving either dietary restriction or 2-DG alone, indicating a synergistic interaction between the drug and the diet. The results suggest that management of malignant astrocytoma with restricted ketogenic diets could be enhanced when combined with drugs that inhibit glycolysis.

## Background

Malignant astrocytomas represent a leading cause of cancer-related death [1-4]. The inability to effectively manage these tumors has been due in part to the unique anatomical and metabolic environment of the brain that prevents the complete resection of tumor tissue and impedes the delivery of therapeutic agents [5]. In contrast to normal brain cells, which can metabolize both glucose and ketone bodies for energy, brain tumors have a reduced capacity to metabolize ketone bodies and, like most malignant tumors, depend heavily on glycolysis for their metabolic energy according to the Warburg cancer theory [6-9]. Hence, therapies that can exploit the differences in energy metabolism between normal brain cells and brain tumor cells should be effective for tumor management [5,6,9].

The high fat/low carbohydrate, ketogenic diet (KD) has antiepileptic, anticonvulsant, and other neuroprotective effects in rodent disease models and in humans [10-17]. A reduction of circulating glucose levels coupled with an elevation of circulating ketone levels is thought to underlie the therapeutic effects [13,18,19]. Studies in children and in experimental brain tumor models showed that the KD administered in restricted amounts (KD-R) is effective in managing tumor growth and in extending survival [15,18,20]. The non-metabolizable glucose analogue 2-deoxy-D-glucose (2-DG) is a potent glycolytic inhibitor that can replicate effects of glucose deprivation in normal cells and in cancer cells both *in vitro *and *in vivo *[21-25]. 2-DG is readily transported into cells and is phosphorylated by hexokinase, but cannot be metabolized further and accumulates in the cell. This leads to ATP depletion and the induction of cell-death. In this regard, 2-DG has been described as a CR-mimetic, a drug that mimics some aspects of calorie restriction [21,26]. Treatment of cancer patients with relatively high doses of 2-DG (greater than 200 mg/kg) was largely ineffective in managing tumor growth [27-29]. Side effects of 2-DG included elevated blood glucose levels, progressive weight loss with lethargy, and behavioral symptoms of hypoglycemia [23,27-29]. Reports that the ketogenic diet could be neuroprotective against hypoglycemic injury [12,16], and that it could also inhibit brain tumor growth by reducing glucose metabolism, suggest that combining the KD-R with low doses of 2-DG (e.g. 25 mg/kg BW) might improve the efficacy of the diet as an anticancer therapy. In the current study, the effects of a KD-R supplemented with a low dose of 2-DG were examined in adult C57BL/6J mice bearing the syngeneic CT-2A malignant astrocytoma grown orthotopically.

### Mice and Experimental Astrocytoma

Mice of the C57BL/6J (B6) strain were obtained from the Jackson Laboratory (Bar Harbor, ME, USA) and were propagated in the Boston College Animal Care Facility as previously described [30]. Adult male mice (~14 weeks of age) were used in this study and were housed individually in plastic cages with filter tops containing Sani-Chip bedding (P.J. Murphy Forest Products Corp., Montville, NJ, USA). The syngeneic malignant mouse astrocytoma was implanted into the cerebral cortex as previously described [31]. The procedures for animal use were in strict adherence to the NIH Guide for the Care and Use of Laboratory Animals and were approved by the Institutional Animal Care Committee at Boston College. Other husbandry conditions were as previously described [30].

### Dietary Regimens, Body Weight, and Food Intake Measurements

Two types of dietary regimens were employed in the study: the standard PROLAB RMH 3000 chow diet (SD) (Lab Diet, Richmond, IN, USA) and the lard-based ketogenic diet (KD) (Zeigler Bros., Inc., Gardners, PA, USA). All mice received PROLAB RMH 3000 chow prior to tumor implantation. The SD contained a balance of mouse nutritional ingredients and delivers 4.1 kcal g^-1 ^gross energy, where fat, carbohydrate, protein, and fiber comprised 55, 520, 225, and 45 g kg^-1 ^of the diet, respectively. The KD also contained a balance of mouse nutritional ingredients. According to the manufacturer's specification, the KD delivers 7.8 kcal g^-1 ^gross energy, where fat, carbohydrate, protein, and fiber comprised 700, 0, 128, and 109 g kg^-1 ^of the diet, respectively. The fat in this diet was derived from lard and the diet had a ketogenic ratio (fats: proteins + carbohydrates) of 5.48:1. Body weight and food intake of all mice was recorded daily (1:00 PM – 3:00 PM). Water was provided *ad libitum *for all mice.

**Figure 1:**
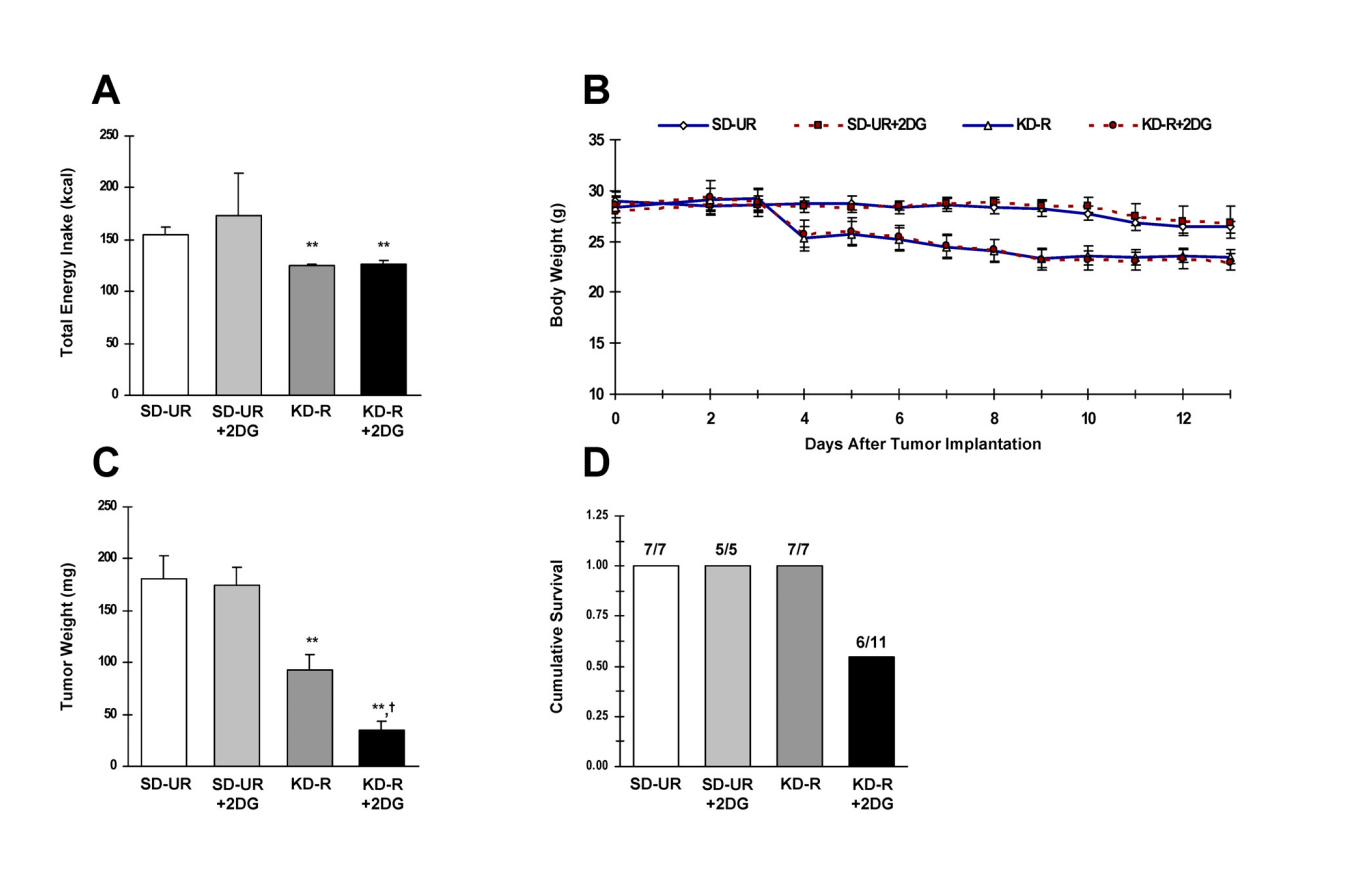
**Influence of the restricted ketogenic diet with or without 2-DG on total energy intake (**A**), body weight (**B**), tumor growth (**C**), and on cumulative survival (**D**) in mice bearing the orthotopically implanted CT-2A malignant astrocytoma.** All mice were fed the standard high carbohydrate rodent diet in UR amounts for the first 3 days after tumor implantation prior to their separation into one of four diet groups (n = 5–11 mice/group) fed either SD-UR or a KD-R with or without 2-DG (25 mg/kg) for 10 days. The four groups were matched for body weight. 2-DG was initiated 6 days after tumor implantation and was continued for 7 days (**B **&**C**). As shown in (**B**), the feeding paradigm for the KD-R and KD-R+2-DG groups was designed to reduce body weights by ~20% relative to values recorded before the diet was initiated (3 days after tumor implantation). The average total energy intakes in (**A**) represent the number of kcals consumed by the indicated group over the dietary treatment period (day 3 to day 13). All values are expressed as the mean ± S.E.M. In (**A **&**C**), average values for the indicated group are significantly less than the average value for the SD-UR group at ** P < 0.01. The mean value for the KD-R+2DG group is significantly lower than the mean value for the KD-R group at † P < 0.01. No significant differences were observed between the SD-UR and SD-UR+2DG groups throughout the study. For (**D**), the number of tumor-bearing mice that were alive in each group at the conclusion of the study is listed as a ratio above each solid vertical bar (e.g. the "6/11" indicates that 6 of the 11 original mice were alive at the end of the study in the associated group).

All mice were fed the standard diet unrestricted for the first 3 days after tumor implantation. They were then separated into one of four diet groups fed either the standard diet in unrestricted amounts (SD-UR) or a KD-R with or without 2-DG (25 mg/kg) for 10 days. The four groups were matched for body weight (~28.8 g) prior to the initiation of the dietary regimens. Low dose treatment with 2-DG was initiated 6 days after tumor implantation and was continued for 7 days (Fig. 1B & Fig. 1C). The feeding paradigms for the KD-R and KD-R+2-DG groups were designed to reduce mouse body weights by ~20% relative to values recorded before the diets were initiated (3 days after tumor implantation). All mice were euthanized 13 days after tumor implantation.

## Results and discussion

Our goal was to determine if low doses of 2-DG, when administered together with the KD-R, might produce synergistic effects. As our preliminary results showed that 2-DG at doses exceeding 250 mg/kg produced adverse effects to include weight loss, anorexia, and death (data not shown), we chose 2-DG at a dose of 25 mg/kg because this dosage did not alter food intake or body weight in tumor-bearing mice fed the KD-R compared with mice fed SD-UR. The therapeutic effects of dietary restriction arise largely from caloric restriction *per se *and not from the restriction of any specific dietary component such as proteins, vitamins, mineral, fats, or carbohydrates [15,32-34]. We did not include an unrestricted KD (KD-UR) group or a KD-UR+2-DG group in the study because we previously showed that the rate of tumor growth in mice fed an unrestricted KD is similar to that of mice fed SD-UR [15]. Moreover, unrestricted feeding of either the KD or standard high carbohydrate diets maintains high glucose levels, which provokes tumor angiogenesis and growth [5,32,35,36]. Restricted KDs maintain higher circulating ketone body levels and equally low glucose levels as restricted high carbohydrate standard diets [13,18]. Administration of the KD in restricted amounts also reduces adverse effects of the diet's high fat content, as fats are rapidly converted to ketone bodies for tissue energy metabolism [13]. Hence, we consider a restricted KD more therapeutic for brain cancer management than restricted high carbohydrate/protein diets. In contrast to 2-DG, which primarily reduces glycolysis through inhibition of hexokinase activity, dietary energy restriction acts as a broad spectrum inhibitor of multiple metabolic and signal transduction pathways without producing adverse effects [5].

Total energy intake was similar over the dietary treatment period (between 3 and 13 days after tumor implantation) in the SD-UR+2-DG and SD-UR groups, but more variability was noticed in the SD-UR+2-DG group (Fig 1A). Energy intake was similar between the KD-R and KD-R+2-DG groups over the dietary treatment period. Body weights were reduced to a similar degree (~20%) in the two groups fed the KD-R (Fig. 1A & Fig. 1B). We previously showed that matching body weights within control and treatment groups was absolutely critical for correct data interpretation [13,18]. In the current study, we found that administration of a low-dose of 2-DG had no significant affect on either body weight or on CT-2A tumor growth in mice fed the SD-UR (Fig. 1B & Fig. 1C). These results contrast with those of Zhu et al., who showed that administration of low dose 2-DG in an unrestricted standard high carbohydrate diet could reduce mammary tumor growth in rats [21]. The differences in the results between the two studies could be attributable to the different rodent species used, the mode of drug delivery, or to the location of the tumor (brain vs. subcutaneous flank).

We found that the average tumor weights for both the KD-R and KD-R+2-DG groups were significantly lower than those for the SD-UR group (48% and 80%, respectively) (Fig. 1C). These observations are consistent with prior studies showing that the KD-R can significantly reduce orthotopic CT-2A tumor growth [15,18], and also provide novel evidence that a low dose of 2-DG could act synergistically with the KD-R to further reduce growth in this mouse astrocytoma. The gross appearance of the mice bearing the CT-2A tumors in the UR and R groups in this study was similar to those that we showed previously for the restricted standard and ketogenic diets [32]. While tumor wet weight was reduced more in the KD-R + 2-DG group than in the KD-R group, facial appearance and skull size was similar in these groups. It is necessary to mention, however, that the combined therapy produced adverse effects on mouse health and vitality. Several mice in the KD-R+2-DG group died while the survivors appeared more lethargic than the mice in the KD-R group (Fig. 1D). We suggest that energy stress was greater in mice receiving the drug/diet combination than in mice receiving either dietary restriction or 2-DG alone. Nevertheless, the results show that 2-DG in combination with the restricted ketogenic diet was synergistic with regard to tumor growth and that potential adverse effects might be reduced with adjustments in either drug concentration or in diet restriction. Further studies will be needed to test this hypothesis.

## Conclusion

Our findings provide novel evidence that the KD-R supplemented with a low dose of 2-DG was effective in reducing intracerebral tumor growth to a greater extent than was either 2-DG or the KD-R administered alone, suggesting a synergistic interaction between the drug and the diet. Although health and vitality were not as strong in the drug/diet group than in the KD-R group, adjustments in either 2-DG concentration or in dietary restriction could mitigate health detrimental effects. Our results suggest that combining drugs that inhibit glycolysis with restricted ketogenic diets could enhance inhibition of malignant astrocytoma growth.

## Competing interests

The authors declare that they have no competing interests.

## Authors' contributions

JM designed the study, performed the research, analyzed the data, and drafted the manuscript; PM helped design the study, and she also provided helpful comments during the drafting of this manuscript; TNS conceived of the initial study, provided support for the research, and helped to prepare the manuscript. All authors have read and approve the final version of the manuscript.
